# Differential toxicity and venom gland gene expression in *Centruroides vittatus*

**DOI:** 10.1371/journal.pone.0184695

**Published:** 2017-10-04

**Authors:** Thomas McElroy, C. Neal McReynolds, Alyssa Gulledge, Kelci R. Knight, Whitney E. Smith, Eric A. Albrecht

**Affiliations:** 1 Department of Ecology, Evolution and Organismal Biology, Kennesaw State University, Kennesaw, GA, United States of America; 2 Department of Biology and Chemistry, Texas A&M International University, Laredo, TX, United States of America; 3 Department of Molecular and Cellular Biology, Kennesaw State University, Kennesaw, GA, United States of America; Instituto Butantan, BRAZIL

## Abstract

Variation in venom toxicity and composition exists in many species. In this study, venom potency and venom gland gene expression was evaluated in *Centruroides vittatus*, size class I-II (immature) and size class IV (adults/penultimate instars) size classes. Venom toxicity was evaluated by probit analysis and returned ED_50_ values of 50.1 μg/g for class IV compared to 134.2 μg/g for class I-II 24 hours post injection, suggesting size class IV was 2.7 fold more potent. Next generation sequencing (NGS and qPCR were used to characterize venom gland gene expression. NGS data was assembled into 36,795 contigs, and annotated using BLASTx with UNIPROT. EdgeR analysis of the sequences showed statistically significant differential expression in transcripts associated with sodium and potassium channel modulation. Sodium channel modulator expression generally favored size class IV; in contrast, potassium channel modulators were favored in size class I-II expression. Real-time quantitative PCR of 14 venom toxin transcripts detected relative expression ratios that paralleled NGS data and identified potential family members or splice variants for several sodium channel modulators. Our data suggests ontogenetic differences in venom potency and venom related genes expression exist between size classes I-II and IV.

## Introduction

Scorpions are well-known, venomous arthropods (Class: Arachnida, Order: Scorpiones) that live in a wide variety of habitats. The genus *Centruroides* (bark scorpion) is commonly found in North America, with habitat ranges from Nebraska to southern Texas [1]. As a nocturnal predator, it uses pedipalps alone or in combination with a venomous sting to feed or defend against a wide range of hexapod and arachnid organisms [2, 3, 4].

Venom is important to all scorpion species for feeding and defense, and the composition of the venom can affect predator-prey interactions [2,5]. Scorpion venom consists of neurotoxins, proteases, and cytotoxic peptides, which are generally classified into disulfide-bridged or non-disulfide-bridged peptide (NDBP) groups [6,7,8]. Biochemical studies characterizing *Centruroides* venom composition have identified several disulfide-bridged proteins, including those functionally known as neurotoxins [9–12]. Neurotoxins, such as sodium and potassium channel modulators, can make up a large percentage of total venom proteins in *Centruroides* venom, suggesting that they are important toxic components [7]. Less is known about non-disulfide bridge peptides found in scorpion venom, however, the activity of these peptides involve pro-inflammatory, antimicrobial and hemolytic activity [9].

Investigations examining proteomic or genomic profiles of individual scorpion venom glands have resolved additional layers of complexity [13]. Several factors such as geographical location, venom synthesis rates and foraging behavior may contribute to reported variability. Geographically separate adult scorpions of the same species contained overlapping but not identical venom composition signatures [14–17]. For example, individual mass spectra of *P*. *transvaalicus* venom showed the relative intensities of individual peaks vary, but the peaks clustered in two major groups separated by a *m/z* range devoid of peptides [18]. This information suggests venom signatures are the same, but differ in intensity of proteomic expression. In contrast, *Mesobuthus gibbosus* venom collected and analyzed by SDS PAGE showed only one band in common between 8 individuals [19]. Other elements such as stimulation frequency or biosynthesis rates appears to influence venom composition, as evident by observations that venom clarity and toxicity changes during successive collection [20]. A study monitoring the venom composition and toxicity of *P*. *transvaalicus*, showed the synthesis of different venom peptides occurs at varying rates. The venom produced 1–2 day after milking was less toxic to crickets compared to venom produced 8 days post milking [21]. This suggests variability may be connect to habitat niche, where foraging behaviors would dictate stinger usage and prey selection. For example, Edmunds et. al., demonstrated that stinger usage in *Hadrurus spadix* was associated with the size and activity level of the prey [5].

Growing evidence from field and laboratory studies suggest ecological and developmental factors affect the composition and toxicity of venom produced by a variety of animals [22–25]. In scorpions, Pucca et. al., reported diet changes altered the proteomic profile and hyaluronidase activity of *Tityus serrulatus* venom [26]. However, details regarding the influence of diet on scorpion venom composition are limited and information regarding the influence diet on venom composition comes from other venomous animals such as snakes. Reports suggest the variation in the *Calloselasma rhodostoma* (Malayan pit viper) venom parallels changes in diet [23] and *Pelias* vipers that preferentially consumed insects possessed greater toxicity towards crickets, compared to those preying on lizards and mice [27]. Similarly, selective consumption of prey appears to develop differential toxicity in the venom of *Echis carinatus* (saw scaled vipers). The venom of *E*. *carinatus* arthropod specialists, induced death and incapacitation faster in scorpions than the species known to prey on vertebrates [28].

In the same way, data from venom producing species has been used to evaluate ontogenetic contributions to venom gland gene expression and venom composition [29,30]. For example, changes at the transcriptome level in toxin expression profiles across developmental stages has been identified in the Central American Rattlesnake *Crotalus simus simus* [31], suggesting that an increase in venom potency can increase predator effectiveness or, alternatively, an increase in prey resistance could decrease prey capture success of the predator. The selection of specific venom proteins due to ontogenetic factors may indirectly develop dietary specialists [32]. Another example of ontogenetic variation was reported in *Conus ebraeus*. The expression of genes functionally associated with venom production changed in accordance with growth and diet in *Conus ebraeus*, suggesting variation of venom composition parallels dietary shifts as the organism grows [25]. Although more details are immerging regarding the extent external factors control the genomic mechanisms involved in venom production, specific information pertaining to *C*. *vittatus* is limited. Ecological studies have been used to understand *C*. *vittatus* feeding behaviors [33–35, 4], but detailed information about *C*. *vitattus* venom composition, venom gland gene expression or toxicity has not been reported. Our goal in this study was to determine if ontogenetic differences exist in *Centruroides vittatus* by evaluating venom toxicity and venom gland gene expression in different size classes (class IV and class I-II).

## Material and methods

### Venom collection

**Fig 1:**
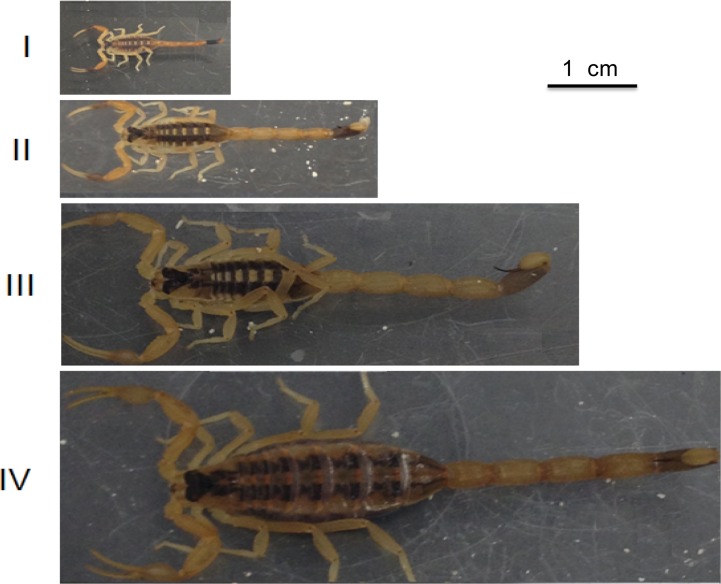
*Centruroides vittatus* size classes. Size class determined by measuring scorpion length from prosoma to the posterior of the mesosoma.

Guadalupe and Lilia Martinez Foundation granted permission to do fieldwork and collect arthropods including Centruroides vittatus at La Union Ranch. *Centruroides vittatus* were collected in two locations Texas A&M International University campus in Laredo, Texas (27°35’ N, 99°26’ W) and La Union Ranch at San Ygnacio, Texas (27°7’ N, 99°19’), sorted by size class, housed individually. Size was determined as described by Polis & McCormick, 1990 [33]. Briefly, scorpions were measured from the anterior of the prosoma to the posterior of the mesosoma. Size classes were estimated in the field with size class I measured < 5 mm, size class II between 5–10 mm, size class III between 10–15 mm and size class IV measured > 15 mm [33]. Specimen examples are shown in Fig 1. Captured scorpions were watered daily and fed one cricket every two weeks for four months. After four months of captive feeding, venom was collected after inducing a strike to a paraffin covered centrifuge tube. One microliter of phosphate buffered saline (PBS) containing 0.01% SDS was added to the drop of venom on the parafilm, aspirated, extracted and stored at -20°C until use. For experimentation, the venom from size class I-II (immature) and size class IV (adults and penultimate instars), were separately pooled. Size class III scorpions were omitted from this study because it was difficult to assign adult or immature status to these individuals. Pooling was necessary because of the limited amount of venom collected from each individual. The total protein concentration for each of the pooled venom samples was determined by Lowry method [36].

### Toxicity assay

The biological activity of collected venom was measured in crickets. Three groups containing 8 crickets per group (randomly assigned to each group) were injected with 20, 65, or 130 μg/g venom from size class IV. Venom dilutions were injected intrathoracically between the second and third pairs of legs of each cricket using a 10-μl glass syringe (Hamilton Company, USA). All doses were mass (μg/g body weight) adjusted. The control group was injected with 1.0 μl PBS. This procedure was repeated using pooled venom from size class I-II. At 1, 2, 3, 4 and 24 hours following injection, the state of each cricket was recorded to the nearest hour using the criteria of Boeve et al. [37]. Behavioral states were defined as (1) dead (unable to right self or motionless); (2) unable to right (UTR) self, but displaying leg movements; or (3) no effect (normal behavior). Paralysis or death are indistinguishable from each other. An effective dose was considered any dose that altered the cricket’s ability to move including paralysis. Results were calculated as a percentage of insects dead, unable to right when placed on the back, or no effect. ED_50_ was calculated using Probit analysis by Finney (1952) with SPSS 24.0 software [38].

### RNA isolation and cDNA preparation

*C*. *vittatus* scorpions were collected and maintained under identical conditions to those used for venom toxicity experiments. The telsons from 9 size class IV and 20 size class I-II scorpions were surgically removed and weighed, 4 days post venom extraction. The total RNA was extracted with Trizol [39]. Briefly, each telson was harvested and immediately ground with Trizol. Individual telson samples were pooled within each size class before further processing. After Trizol isolation, the two samples of total RNA (one pool of size class IV and one pool for size class I-II) were DNAse treated and cleaned up using the Qiagen RNeasy Mini Kit. Total RNA was evaluated for integrity using 2100 Expert Agilent bioanalyzer. Four micrograms of total RNA were used to prepare each cDNA library following the TruSeq protocol of stranded mRNA Sample Preparation from Illumina. [TruSeq® RNA Sample Preparation v2 Guide, 2014].

### Sequencing and assembly

Two paired-end reads per biological sample were generated on Illumina Hiseq 2500 following manufacturer's protocol and provided >5 million reads for class IV and >4 million reads for class I-II with average sequence lengths of 100 base pairs. Raw sequencing reads are archived under SRA study number SRP101778 and BioProject accession PRJNA378557. More than 87% of the bases have the error rate less than 1/1000 (Phred score >30) and the potential content of the sequencing adaptor in the raw reads is less than 3% for the two samples. Raw reads were assembled into 36,795 contigs using Trinity. The total number of assembled bases is 24,959,975 with N50 equal to 6,579 nt.[40]. During the assignment of gene ontology, assembled contigs were aligned to annotated peptides of scorpions from UniProt using BLASTx with cutoff e-values < 10e-5. Additional cross-referencing of selected annotated transcripts was done in Venom Zone (http://venomzone.expasy.org/). Bowtie2 was used to map reads to assembled contigs. The abundance of assembled contigs was estimated using RNA-Seq by Expectation Maximization (RSEM), which provided measurements of expression as raw counts, transcripts per million, and fragments per kilobase of transcript per million (FPKM) [41]. Size class FPKM values generated for each assembled sequence were used to construct FPKM value (IV/I-II) ratios.

### Differential expression statistics

Statistical analysis of differential expression was performed using EdgeR v3.12.1 [42–44]. With n = 1 per group, the biological dispersion (coefficient of variation) was estimated from the common transcripts showing similar expression between size class IV and size class I-II (|log2 Fold change| < 0.1). Results with p values ≤ 0.001 were considered significantly different.

### qPCR

The same total RNA isolated from the venom glands and used to build the transcriptome of size class IV and I-II was used to evaluate the expression of some venom related transcripts identified from our transcriptome. One microgram of total RNA from each pooled sample was reverse transcribed using Qiagen QuantiTect Reverse Transcription Kit, producing cDNA and stored at -20˚C. We used scorpion GAPDH as the housekeeping gene, and created a cDNA standard from a mixture of class IV and class I-II cDNAs. Quantitative PCR was preformed using a Roche 480 LifeCycle with SYBR green dye. For each duplicate reaction, 1.0 μl of diluted cDNA was combined with SYBR green PCR Master Mix (Thermofisher, CA) and assayed in 96 well optical grade PCR plates containing gene specific primers (IDT Inc, Coralville, IA). Melting curve analysis confirmed single amplicon (non-contaminated) products were synthesized. Duplicate reactions were repeated until standard deviations were less than 0.25. Relative gene expression was calculated by the 2-ΔΔC_T_ method [45]. We used the relative gene expression of each transcript (transcript/GAPDH) to construct expression ratios between size classes: (transcript IV /GAPDH IV) / (transcript I-II/ GAPDH I-II).

## Results

### Toxicity assay

**Fig 2:**
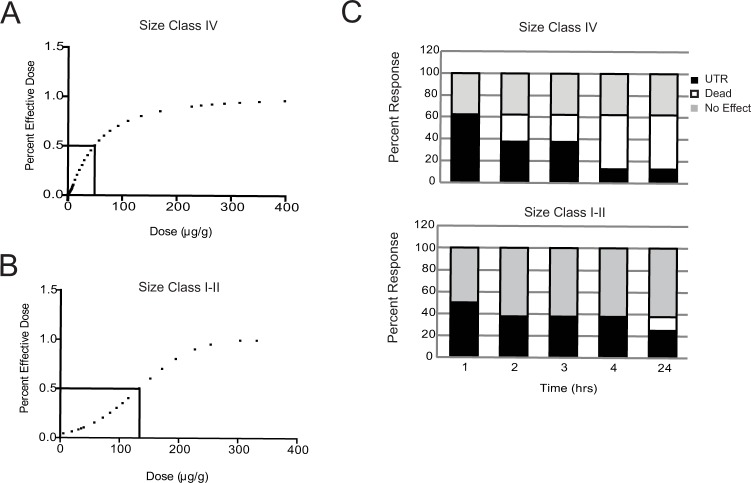
Effective dose response curves. Live crickets were injected with, PBS (0), 20, 65, or 134.2 μg/g crude venom. (A) ED50 curves for class IV after 24 hours were 50.1 μg/g (p<0.01) (B) ED50 curves for class I–II after 24 hours were 134.2 μg/g (p <0.02). (C) Temporal responses to 130 μg/g venom were monitored every hour for 4 hours then again at the 24 hour time point. Class I—II venom induced the inability to right (UTR) in 37.5–50.0% of the individuals during early time periods (1–4 hours). Twenty-four hours post stimulation one death (12.5%) was recorded with the remaining individuals unaffected or UTR. Class IV venom demonstrated greater potency inducing UTR response in 62.5% after 1 hour, and a significant shift from UTR to death overtime (Black (UTR) to White (Dead)) (n = 8 per group).

The toxicity of size class IV and I-II venom was investigated by calculating the median effective dose (ED50) in crickets 24 hours after treatment. Behavioral states were defined as (1) dead (unable to right self or motionless); (2) unable to right (UTR) self, but displaying leg movements; or (3) no effect (normal behavior). Paralysis or death are indistinguishable from each other. An effective dose was considered any dose that altered the cricket’s ability to move including paralysis. Control crickets injected with 1.0 μl of phosphate buffer solution were not affected after 24 hours and demonstrated normal behavior. Probit analysis reported ED_50_ values of 50.1 μg/g for size class IV compared to 134.2 μg/g for size class I-II scorpions; 24 hours post injection (Fig 2A and 2B). Size class IV scorpion venom had a 2.7-fold higher potency than size class I-II scorpion venom. Significantly different ED_50_ dose curves supported differences in venom potency between IV and I-II size classes, with slope values of 2.65 and 0.038, respectively. As a part of the toxicity evaluation, we also monitored the time taken for crickets to become incapacitated following the highest venom dose (Fig 2C and 2D). The temporal responses to 130 μg/g of class IV and class I-II venom in crickets were monitored every hour, for 4 hours; with a final assessment 24 hours post injection. Injections with pooled class I-II venom, found 37.5% of the crickets were unable to right self (UTR) 4 hours post injection, while the remaining 62.5% were unaffected. In contrast, 62.5% of the crickets injected with class IV venom were deceased or incapacitated after 4 hours.

### Analysis of assembled sequences

**Fig 3:**
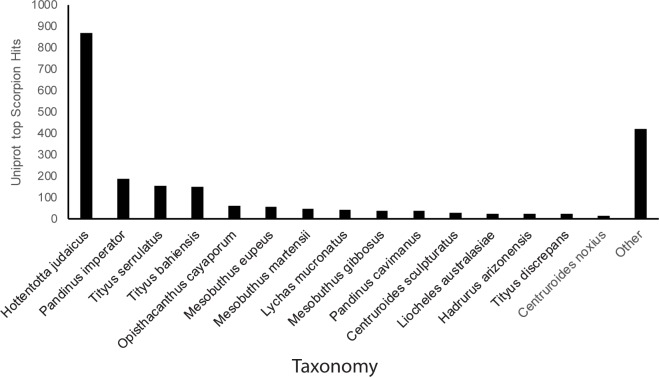
Distribution of taxonomic identification within scorpion species. Taxonomic identification of 2,642 annotated transcripts with species identification hits >10e^-5^. Taxa higher than the order Scorpiones represented in “other” category.

Next generation sequencing technology was used to characterize the venom gland transcriptome of class IV and class I-II *Centruroides vittatus*. Due to the small size of the species, and thus the very limited amount of venom gland RNA, two pooled samples were used, combining 9 size class IV scorpions, and 20 size class I and II scorpions. Even using this method, the final size class I-II sample pool had just enough RNA to allow 2 runs of RNA sequencing and qPCR follow-up. Ilumina run statistics reported an error rate below 0.01 for 92% of the class IV reads compared to 94% of the class I-II reads. Sequencing reads from both sample groups were assembled into 36,795 total contigs using Trinity, which were queried against the UniProt database using BLASTx. This analysis returned 2,642 annotated transcripts with e-values < 10e^-5^. The remaining contigs (non-annotated) produced alignments with e-values > 10e^-5^ or no alignment at all. The 2,642 transcripts that met our e-value criteria were classified by species hit frequency. Results indicated that 2,028 transcripts (77%) could be mapped to scorpion species and 184 (7%) could be mapped to higher scorpion taxa, while the remaining 420 (16%) mapped to taxonomic levels higher than the Order Scorpiones and were designated as “other” (Fig 3).

RSEM analysis was applied to our 36,795 sequences, followed by differential gene expression with EdgeR. A list consisting of these sequences (annotated transcripts and unannotated contigs) was sorted first by decreasing class IV FPKM values, then sorted by decreasing class I-II FPKM values. The top 25 sequences from each sorted list were combined, followed by the removal of duplicates; this resulted in 29 individual sequences (Table 1). From this list, 8 sequences had a 2-fold or greater expression difference between size class IV and I-II. The sum total of normalized FPKM values for size class IV compared to size class I-II was 636434 and 553591, respectively.

**Table 1:** Top 29 expressed sequences identified from the UniProt database.

Annotation	Normalized FPKM Size Class IV	Normalized FPKM Size Class I-II	Ratio (IV/I-II)	ID
Putative uncharacterized protein	167181	182510	0.92	UniRef90_C9X4G3
*Antimicrobial NDBP 6	93911	80253	1.17	AHZ63125.1
Bradykinin-potentiating peptide	57237	55951	1.02	UniRef90_C9X4J0
*Toxin Css39.8	**47435**	**1947**	**24.36**	**UniRef90_B7FDP2**
*α-toxin Cn12	**46266**	**13678**	**3.38**	**UniRef90_P63019**
*Neurotoxin LmNaTx30	**25365**	**7221**	**3.51**	**UniRef90_P0CI52**
*Venom protein 164	14905	9359	1.59	UniRef90_P0CJ13
*Venom protein VP6	13296	19503	0.68	UniRef90_F1CJ08
Actin-57B	12315	10035	1.23	UniRef90_P53501
Uncharacterized protein	11067	10628	1.04	UniRef90_F1CJ95
Cytochrome c oxidase subunit 3	10568	14345	0.74	UniRef90_B2CKW2
*Toxin CsEv1	10500	10620	0.99	UniRef90_P01492
*β-toxin CeII8	9798	7245	1.35	UniRef90_P0CH40
Metalloserrulase 3	8907	4591	1.94	UniRef90_A0A076L3I0
**Uncharacterized protein**	**8693**	**2**	**4282.19**	**n/a**
**Phi-buthitoxin-Hj1a**	**8472**	**17088**	**0.50**	**UniRef90_F1CIZ6**
Uncharacterized protein	8458	6473	1.31	UniRef90_F1CJ95
**Uncharacterized protein**	**7912**	**15418**	**0.51**	**n/a**
*Neurotoxin Cex13	**7537**	**2929**	**2.57**	**UniRef90_Q68PG2**
Ferritin	7372	5722	1.29	UniRef90_C5J8A9
Puniative 60s ribosomal protein I32	7109	7212	0.99	n/a
Elongation factor 1-alpha	6939	7479	0.93	UniRef90_Q9BNU1
*α-KTx 28.1	**6889**	**12553**	**0.55**	**UniRef90_R4GUQ3**
60s ribosomal protein	5722	6657	0.86	UniRef90_C9X4I3
Ubiquitin-60S ribosomal protein L40	5665	6531	0.87	UniRef90_P14794
Uncharacterized protein	4681	6514	0.72	n/a
Putative uncharacterized protein	4140	2809	1.47	UniRef90_C9X4H4
Hypothetical secreted protein	4025	5054	0.80	UniRef90_F1CJ08
*Toxin Pg8	**2878**	**6080**	**0.47**	**UniRef90_B7SNV8**

The results sorted by greatest expression (FPKM) in the Class IV category; FPKM; fragments per kilobase of transcript per million.

^a^ Transcripts identified with (*) indicate venom related transcripts

^b^ Transcript rows identified with bold font p< 0.001 as determined by EdgeR analysis, and FPKM difference of ≥ 2.0-fold

The 29 sequences presented in Table 1 include several transcripts that are annotated to genes defined as venom toxins (indicated with asterisk) such as Toxin Css 39.8, α-toxin Cn12, Neurotoxin LmNaTx30, and Venom protein 164. Interestingly, the FPKM expression sum totals of transcripts specific to venom toxins favored class IV, with FPKM totals equal to 297,551 for size class IV compared to 203,006 for size class I-II. Although total toxin expression favored size class IV, individual toxin expression varied between size classes. For example, Toxin Css39.8, α-toxin Cn12, Neurotoxin LmNaTx30, Neurotoxin Cex13, and an uncharacterized protein show FPKM values favoring size class IV scorpions by more than 2-fold, supported by significant p values (<0.001), calculated with EdgeR. In contrast, Toxin Pg8 had a greater than 2-fold expression favoring the size class I-II group. Other annotated transcripts listed in Table 1 function in translation (e.g., Elongation factor 1-alpha, 60s ribosomal protein I32), cytoskeletal (e.g., Actin 57B), and cellular or inflammatory processes (e.g., cytochrome C oxidase S3, ferritin, bradykinin-potentiating peptide). Several highly expressed transcripts, such as bradykinin-potentiating peptide and metalloserrulase, are well described in other scorpion species [46–48]. Eight uncharacterized/ ‘hypothetical' proteins were also identified and potentially represent new venom components

### Differential expression of venom related transcripts

We queried the 2,642 annotated transcripts against UniProt and Venom Zone database for the terms ‘venom’ and ‘toxin’ to further explore differential gene expression of venom related transcripts resulting in a list of 70 transcripts (S1 Table). Table 2 shows a subset of 46 transcripts from this list grouped by protein family/protein function. Each transcript has the FPKM ratio of IV/I-II shown, where ratios greater than 1.0 represent higher size class IV expression and values below 1.0 favor size class I-II expression. Analysis with EdgeR was included (see Table 2), and transcripts that have both a ratio of ≥2.0 or ≤0.5, and a p value of <0.001 are indicated.

**Table 2:** Transcripts associated with venom and venom toxicity.

Annotation	Normalized FPKM Size Class IV	Normalized FPKM Size Class I-II	Ratio (IV/I-II)	ID
***Calcium Signaling Modulator***				
Cysteine-rich venom protein LEI1-like	196	180	1.09	UniRef90_A0A0C9RP98
Venom allergen 5	145	110	1.32	UniRef90_A0A0C9RP88
***K Channel Modulators***				
**Phi-buthitoxin-Hj1a**	**8472**	**17088**	**0.50**	**UniRef90_F1CIZ6**
α-KTx 28.1*	**6889**	**12553**	**0.50**	**UniRef90_R4GUQ3**
pMeKTx30-1*	**3082**	**5440**	**0.57**	**UniRef90_A0A088DAF5**
pMeKTx21-1	2413	2579	0.94	UniRef90_A0A088D9V0
α-KTx 10.1*	**598**	**729**	**0.82**	**UniRef90_O46028**
***Na Channel Modulators***				
Toxin Css39*	**47435**	**1947**	**24.36**	**UniRef90_B7FDP2**
α-toxin Cn12*	**46266**	**13678**	**3.38**	**UniRef90_P63019**
**Neurotoxin LmNaTx30**	**25365**	**7221**	**3.51**	**UniRef90_P0CI52**
Toxin CsEv1	10500	10620	0.99	UniRef90_P01492
β-toxin CeII8*	**9798**	**7245**	**1.35**	**UniRef90_P0CH40**
Neurotoxin Cex13*	**7537**	**2929**	**2.57**	**UniRef90_Q68PG2**
Toxin Pg8*	**2878**	**6080**	**0.47**	**UniRef90_B7SNV8**
Lipolysis-activating peptide 1-α*	**1415**	**1249**	**1.13**	**UniRef90_P0CI44**
Toxin Acra III- 1 (long)*	**1337**	**98**	**13.64**	**UniRef90_P0C298**
Toxin Acra III- 2 (long)*	**1071**	**193**	**5.55**	**UniRef90_B8XH01**
Toxin Acra III- 2 (short)*	**984**	**674**	**1.46**	**UniRef90_B8XH01**
Toxin Acra III- 1 (short)*	**796**	**107**	**7.44**	**UniRef90_P0C298**
Beta-insect toxin AaBTxL1	290	168	1.72	UniRef90_Q4LCS8
***Anti-Microbial***				
Antimicrobial NDBP 6*	**93911**	**80253**	**1.17**	**AHZ63125.1**
4 kDa defensin	440	423	1.04	UniRef90_P56686
**Antimicrobial peptide TsAP-2**	**277**	**104**	**2.66**	**UniRef90_S6D3A7**
**Ponericin-W-like 32.1**	**103**	**14**	**7.36**	**UniRef90_P0CI91**
***Protease/ Hydrolytic Enzymes***				
Metalloserrulase 3	8907	4591	1.94	UniRef90_A0A076L3I0
Trypsin-like S1/S6 peptidase	1197	1270	0.94	UniRef90_A0A0C9S383
AbCp-11 (colipase-like)	972	1010	0.96	UniRef90_C5J8A3
Venom leucine aminopeptidase	972	812	1.20	UniRef90_E4VP13
Cathepsin F-like cysteine peptidase	374	442	0.85	UniRef90_U6JPB2
**Serine proteinase stubble**	**296**	**639**	**0.46**	**A0A087T9S0**
Chitinase 3	275	215	1.28	UniRef90_A0A0C9RPB5
**Metalloendopeptidase**	**164**	**17**	**9.65**	**UniRef90_U6JRL7**
**Venom protein AbVp 1 (M13 peptidase)**	**145**	**7**	**20.71**	**UniRef90_E4VP09**
Trypsin-like serine peptidase 3 protein	143	78	1.83	UniRef90_U6JRJ9
**Chitinase**	**45**	**130**	**0.35**	**UniRef90_A0A0C9S0K3**
***Protease Inhibitor***				
Venom protein 302	1855	2428	0.76	UniRef90_A0A0C9RPA6
Venom protein 9	224	427	0.52	UniRef90_E4VP39
Serpin B6-like	110	127	0.87	UniRef90_A0A0C9S0I9
***Other***				
Venom protein 164	14905	9359	1.59	UniRef90_P0CJ13
Venom protein VP6	13296	19503	0.68	UniRef90_F1CJ08
Venom protein AbVp 9*	**4025**	**5054**	**0.80**	**UniRef90_F1CJ08**
Serin-type endopeptidase	2784	3351	0.83	UniRef90_Q686B4
**Venom protein 29**	**268**	**104**	**2.58**	**UniRef90_P0CJ08**
Fibrinolytic protease	248	299	0.83	UniRef90_A0A0C9QKS2
Hemolectin	116	60	1.93	UniRef90_F1CJ20
Venom protein 214*	**45**	**284**	**0.16**	**UniRef90_P0CJ10**

Transcripts in each functional category sorted by decreasing class IV value.

FPKM; fragments per kilobase of transcript per million.

^a^ Transcripts identified with (*) in annotation column evaluated by qPCR.

^b^ Transcript rows identified with bold font p< 0.001 as determined by EdgeR analysis, and FPKM difference of ≥ 2.0-fold

From the list of 46 transcripts, 13 represent sodium channel modulators. Notably, in the original list of 70 transcripts, several of the annotated sodium channel modulators had multiple transcripts with the same annotation/accession number, suggesting either alternative splicing or highly similar family members. One example is Toxin Acra III-1, which had one long sequence and one short sequence annotated with and identical accession number.

The most abundant venom transcript encoded a sodium channel modulator, Toxin Css 39.8. This transcript also showed a high level of differential expression, registering normalized FPKM values of 47435 for size class IV compared to 1947 in size class I-II, with a IV/I-II ratio of 24.4 and a p value of <1x10^-300^ (Table 2). Phi-buthitoxin Hj1a represented highly expressed potassium channel modulator, with FPKM values of 17088 (I-II) and 8472 (IV), a IV/I-II ratio of 0.496 and a p value of <1x10^-300^. Interestingly, NGS transcript expression for sodium channel modulators favored size class IV (exception included Toxin Pg8 and Toxin CsEv1), while all potassium channel modulator values favored size class I-II, suggesting sodium and potassium channel modulator genes as the potential source of ontogenetic venom toxicity differences (for p values, see S1 Table). Also notable is the fact that while the sodium channel modulators include both inhibitors and activators (at a 2:1 ratio), the potassium channel modulators all fall into one of two classes of channel blockers.

Anti-microbial non-disulfide bond protein (NDBP) 6 recorded the highest size class FPKM values for transcripts associated with venom, yet displayed only a modest difference in differential expression with a IV/I-II ratio of 1.2. Another highly populated group listed in Table 2 includes proteases, which are often involved in distributing venom within the prey.

### qPCR confirmation of venom related transcripts

**Fig 4:**
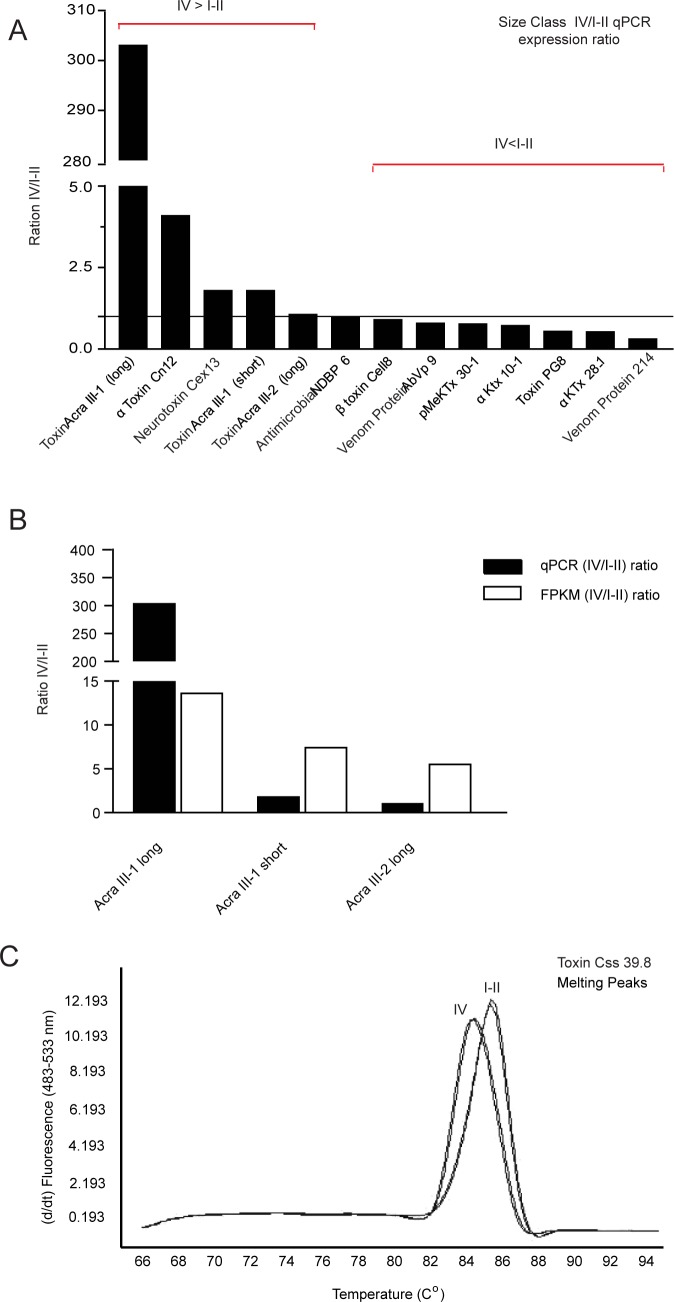
qPCR analysis of select venom transcripts. (A) Transcripts reported as qPCR IV/I-II expression ratios (B) Comparison of Toxin Acra III transcripts by qPCR IV/I-II expression ratio (black columns) and RNA-Seq (FPKM) IV/I-II value ratios (white column). (C) Toxin Css 39.8 melting curve demonstrating two different amplicons in each size class.

We selected 14 differentially expressed transcripts from Table 2 (identified by asterisk) to analyze by qPCR, with 13 analyses shown in Fig 4. Fig 4A depicts the IV/I-II ratio per transcript tested. Similar to Table 2, expression ratios of IV/I-II greater than 1.0 are class IV dominant in expression and values below 1.0 favor size class I-II expression (Fig 4A and 4B). The pattern of expression ratios obtained from qPCR analysis primarily matched Table 2 FPKM IV/I-II values ratios, with ratio differences ≤ 0.78 for 10 of the 13 transcripts examined. The remaining three transcripts displayed the same pattern of expression (IV dominant), however the level of expression and calculated expression ratios from qPCR analysis were substantially different compared to the FPKM IV/I-II value ratios depicted in Table 2. Fig 4B shows a comparison of Toxin Acra III IV/I-II expression ratios, derived from FPKM values (white columns) and qPCR (black columns). Toxin Acra III-1(long) returned a IV/I-II qPCR expression ratio of 303.1, whereas the FPKM (IV/I-II) values gave a ratio of 13.6. Toxin Acra III-1 (short), gave a qPCR expression IV/I-II ratio of 1.8, much lower than the 7.4 FPKM IV/I-II value ratio. Toxin Acra III-2 (long) displayed a qPCR IV/I-II ratio of 1.1, also much lower compared to a FPKM IV/I-II value ratio of 5.5.

The 14th transcript evaluated was Toxin Css 39.8, a known sodium channel modulator. However, qPCR gave two distinct melting curve peaks (84.5°C and 85.8°C), one specific to size class IV (left peak) and one specific to size class I-II (right peak)(Fig 4C). The disparity in the melting curves suggested that size class IV have different amplicons, representing potential alternate splice variants. Therefore, qPCR could not be used to confirm the expression ratio of Css 39.8 between size classes.

## Discussion

*Centruroides vittatus* live in a wide variety of habitats and feed on a wide variety of prey items including intra-guild predation [35, 49, 50]. Differences in venom composition and toxicity is reported in a variety of organisms [24, 32]. These differences are often linked to differential gene expression or post-translational modifications related to environmental factors. However, variation in venom composition can have additional practical factors that contribute to variation including: collection techniques and collection periods. Thus, deciphering the regulation of venom production remains difficult.

In this study, we removed environmental factors to determine if ontogenetic mechanisms contribute to differences in venom toxicity and venom related gene expression. In our experiment, the tested individuals consumed equivalent diets (crickets) for 4 months prior to testing. Individual grouping was based on body lengths. This is, at best, a surrogate of the actual age of the individual. This form of age characterization is useful when an organism’s ontogeny does not have unique developmental markers such as gonad morphological changes. Nonetheless, our results found size class IV crude venom was 2.7 fold more potent than size class I-II venom, suggesting that venom toxicity and, by extension, venom composition differences were independent of foraging behavior and that development stage may influence the composition of *C*. *vittatus* venom.

Scorpions are generalist predators, including *C*. *vittatus* [4, 35, 49]. Therefore, the venom of the scorpion would have to be effective for a wide range of prey taxa. The size of the scorpion can affect foraging and diet [51]. For example, size class IV may control smaller prey using pedipalps, compared to the use of venom for size class I-II scorpions. We observed this behavior in our study during captive feeding. In addition, intra-guild predation is likely greatest for smaller scorpions and may limit foraging behavior [51].

We also documented differential gene expression between size class IV and size class I-II scorpions with transcriptomics and qPCR, providing a quantitative profile of the gland's transcriptome at a specific time point [52, 53]. Transcriptome analysis of each size class found quantitative expression differences in several venom and non-venom transcripts even though diet, environmental conditions, and venom extraction techniques were held constant. Venom transcripts annotated to sodium channel modulators displayed the highest differential expression as depicted in Table 2. Seven of these transcripts showed a greater than 2-fold expression difference in the class IV, and only 1 (Toxin Pg8) showed expression greater than 2-fold in class I-II. This data suggest genes associated with sodium channel modulation display differential ontogenetic expression. Several studies have reported sodium channel modulators as the main source of toxicity in *Centruroides* venom [10, 54]. Our data supports these observations. The antimicrobial peptides (e.g., Antimicrobial peptide TsAP-2 and Ponericin-W-like 32.1) and some of the proteases (e.g., Metalloendopeptidase and Venom protein AbVp 1 (M13 peptidase)) also showed a higher expression in the class IV group. Many of these are likely involved in overcoming prey response to envenomation and mediate venom toxicity by destruction of tissue surrounding the site of envenomation [55,56].

In contrast, 3 of the 5 transcripts associated with potassium channel modulation, and specifically inhibition, (e.g., Phi-Buthitoxin-Hj1a and α-KTx 28.1) exhibited higher expression in size class I-II (Table 2). Scorpion toxin proteins that target potassium channels appear to exert toxicity by reducing nerve cell signal conductance [57, 58]. Several contigs did not align via BLASTx to entries in UniProt and Venom Zone databases, some few did align with entries in the GenBank database; some remained unannotated but exhibited high FPKM values, indicating high levels of expression, and differential expression, as calculated through EdgeR and class IV/I-II ratios. Further investigation will delineate any toxicity function for the protein products of these transcripts.

Selection of statistical analyses is important for proper interpretation of transcriptomic data. EdgeR was chosen as an analysis platform due to small sample size and performance compared other analysis programs (e.g., DEseq, EBseq, NBPseq)(42). This strengthened our selection strategy of a ≥2.0 biological threshold, providing the basis for selecting genes (transcripts) of interest for further analysis.

Considering there was one biological sample per group, the use of statistical analysis was necessarily weak. We utilized qPCR to give a more accurate reflection of gene expression. We identified 14 venom associated transcripts to analyze. Since the venom of *C*. *vittatus* has not been previously characterized, we selected some transcripts that were differentially expressed and some that were not differentially expressed according to our selection strategy. Generally, the relative expression ratios from the qPCR were the same or similar to the ratios reported from the transcriptome analysis.

Within the group of 14 selected transcripts, we discovered two transcriptome alignment artifacts, one in the Toxin Acra III group and one in Css 39.8. All of these genes encode for sodium channel modulators. For Toxin Acra III group we found several transcripts that could represent protein family members or potential splice variants (Fig 4B). Transcriptome analysis indicated that all three of the examined transcripts had an adult dominant expression of greater than 2-fold. However, our qPCR results did not agree. Such disparity may be due to alignment of RNA seq reads to the assembled transcriptome; 100 bp reads may be aligned to highly conserved areas of multiple sequences [40, 41, 59]. Toxin Acra III-1 (long) had much greater expression in the size class IV with a ratio of 303.1-fold, whereas Toxin Acra III-1 (short) and Toxin Acra III-2 both have less than 2-fold difference (1.8 and 1.1 respectively).

Amplification of Css 39.8 gave two distinct peaks in the melting curves, one specific to the class IV and one specific to the class I-II. The difference in the melting curves suggested that the class IV have either a smaller amplicon, or a more A/T rich amplicon, which could mean that an exon was removed or an alternative exon was used. The transcriptome assembly did not give a second (or third) sequence to account for this discrepancy; agarose gel electrophoresis was unable to resolve the difference between the amplicons, suggesting that it may be a small, ~20 base pair exon removal, or be a substituted exon of the same size. Sequencing of the amplicons, as well as sequencing of the genomic DNA will allow us to pinpoint the expression differences of Toxin Css 39.8 between the two size classes.

The observed variability in venom measured in individuals from the same species may be derived from genetic variation among individuals and, likely, plasticity of gene expression in response to the environment. Venom composition, like any quantitative trait, is at once the result of phylogenetic history, species adaptations and local adaptations. Individuals within each population may have molecular mechanisms to change venom composition with respect to developmental age (ontogenetic shift in gene expression) or prey availability (environmentally mediated shift in gene expression). This study represents the first attempt to characterize the venom gland transcriptome of *Centruroides vittatus* and relate it to differences in toxicity between different size classes of the species. More work is needed to resolve the factors that determine venom variability and composition for this species and how this is related to the ecology of the species.

## Supporting information

S1 Table Differential gene expression of 70 venom related genes. (PDF)
