# Supplementary material for: Differential toxicity and venom gland gene expression in Centruroides vittatus
Source: PLoS One. 2017 Oct 4;12(10):e0184695. doi: 10.1371/journal.pone.0184695 (PMC5627916; doi:10.1371/journal.pone.0184695)
Supplement: S1 Table — (PDF) [file pone.0184695.s001.pdf]

S1 Table. Differential gene expression of 70 venom related genes.

Annotation	Normalized FPKM Size Class IV	Normalized FPKM Size Class I-II	IV/I-II Ratios	ID
**Ca Channel inhibitor**				
Cysteine-rich venom protein LEI1-like	196	180	1.1	UniRef90_A0A0C9RP98
Venom allergen 5	124	75	1.7	UniRef90_A0A0C9QKT2
Venom allergen 5	145	110	1.3	UniRef90_A0A0C9RP88
**K Channel inhibitor**				
Phi-buthitoxin-Hj1a	8472	17088	0.5	UniRef90_F1CIZ6
Neurotoxin alpha-KTx 28.1	6889	12553	0.5	UniRef90_R4GUQ3
pMeKTx30-1	3082	5440	0.6	UniRef90_A0A088DAF5
pMeKTx21-1	2413	2579	0.9	UniRef90_A0A088D9V0
Potassium channel toxin meuK1	1314	1909	0.7	n/a
Kunitz-type serine protease inhibitor BmKTT-2	648	506	1.3	UniRef90_P0DJ50
α-KTx 10.1	598	729	0.8	UniRef90_O46028
Kunitz-type serine protease inhibitor BmKTT-2	541	286	1.9	UniRef90_P0DJ50
Kunitz-type serine protease inhibitor BmKTT-2	526	459	1.1	UniRef90_P0DJ50
α-KTx 4.5	472	385	1.2	UniRef90_Q5G8B6
Kunitz-type serine protease inhibitor BmKTT-2	128	198	0.6	UniRef90_P0DJ50
**Na Channel inhibitor**				
Toxin Css39.8	47435	1947	24.4	UniRef90_B7FDP2
Alpha-toxin Cn12	46266	13678	3.4	UniRef90_P63019
Neurotoxin LmNaTx30	25365	7221	3.5	UniRef90_P0CI52
Toxin CsEv1	10500	10620	1.0	UniRef90_P01492
Toxin CsEv1	10250	10284	1.0	UniRef90_P01492
Beta-toxin CeII8	9798	7245	1.4	UniRef90_P0CH40
Neurotoxin Cex13	7537	2929	2.6	UniRef90_Q68PG2
Toxin Pg8	2878	6080	0.5	UniRef90_B7SNV8
Lipolysis-activating peptide 1-α	1415	1249	1.1	UniRef90_P0CI44
Toxin Acra III- 1 (long)	1337	98	13.6	UniRef90_P0C298
Toxin Acra III- 2 (long)	1071	193	5.5	UniRef90_B8XH01
Toxin Acra III- 2 (short)	984	674	1.5	UniRef90_B8XH01
Toxin Acra III- 1 (short)	796	107	7.4	UniRef90_P0C298
Toxin Acra III-2	784	645	1.2	UniRef90_B8XH01
Lipolysis-activating peptide 1-α	543	0	1292.9	UniRef90_E4VP63
Lipolysis-activating peptide 1-α	378	0	759.0	UniRef90_E4VP63
Beta-insect toxin AaBTxL1	290	168	1.7	UniRef90_Q4LCS8
**Anti-Microbial**				
Antimicrobial NDBP 6	93911	80253	1.2	AHZ63125.1
4 kDa defensin	440	423	1.0	UniRef90_P56686
Antimicrobial peptide TsAP-2	277	104	2.7	UniRef90_S6D3A7
Ponericin-W-like 32.1	103	14	7.2	UniRef90_P0CI91
**Protease/ Hydrolytic Enzymes**				
Metalloserrulase 3	8907	4591	1.9	UniRef90_A0A076L3I0
Trypsin-like S1/S6 peptidase	1197	1270	0.9	UniRef90_A0A0C9S383
AbCp-11 (colipase-like)	972	1010	1.0	UniRef90_C5J8A3
Venom leucine aminopeptidase	972	812	1.2	UniRef90_E4VP13
Cathepsin F-like cysteine peptidase	374	442	0.8	UniRef90_U6JPB2
Serine proteinase stubble	296	639	0.5	A0A087T9S0
Chitinase 3	275	215	1.3	UniRef90_A0A0C9RPB5
Cathepsin F-like cysteine peptidase	215	298	0.7	UniRef90_U6JPB2
Metalloendopeptidase	188	177	1.1	UniRef90_U6JRK5
Metalloendopeptidase	164	17	9.4	UniRef90_U6JRL7
Venom protein AbVp 1 (M13 peptidase)	145	7	20.7	UniRef90_E4VP09
Trypsin-like serine peptidase 3 protein	143	78	1.8	UniRef90_U6JRJ9
Chitinase	45	130	0.3	UniRef90_A0A0C9S0K3
**Protease Inhibitor**				
Venom protein 302	1855	2428	0.8	UniRef90_A0A0C9RPA6
Venom protein 9	224	427	0.5	UniRef90_E4VP39
Serpin B6-like	110	127	0.9	UniRef90_A0A0C9S0I9
Serpin B3-like	93	150	0.6	UniRef90_A0A0C9S385
**Other**				
Venom protein 164	14905	9359	1.6	UniRef90_P0CJ13
Venom protein VP6	13296	19503	0.7	UniRef90_F1CJ08
Venom protein VP6	11190	17184	0.7	n/a
Venom protein AbVp 9	4025	5054	0.8	UniRef90_F1CJ08
Serin-type endopeptidase	2784	3351	0.8	UniRef90_Q686B4
Venom peptide meuPep34	3095	2835	1.1	n/a
Venom protein VP6	1124	1167	1.0	UniRef90_F1CJ08
Toxin-like protein TxLP9	892	875	1.0	UniRef90_C5J8B8
Toxin BmTxKS4	539	300	1.8	UniRef90_Q5F1N4
Venom protein 5	449	479	0.9	UniRef90_E4VP54
Venom neuropeptide-3; Orcokinin	435	318	1.4	UniRef90_E4VP55
Venom protein 29	268	104	2.6	UniRef90_P0CJ08
Fibrinolytic protease	248	299	0.8	UniRef90_A0A0C9QKS2
Hemolectin	116	60	1.9	UniRef90_F1CJ20
Hemolectin	112	65	1.7	UniRef90_F1CJ20
Hemolectin	106	39	2.7	UniRef90_H2CYR1
Hemolectin	103	34	3.0	UniRef90_H2CYR1
Venom protein 214	45	284	0.2	UniRef90_P0CJ10
